# Adolescents' screen media entertainment: a quantitative, cross-sectional study

**DOI:** 10.3389/fpsyg.2025.1549080

**Published:** 2025-07-15

**Authors:** Semen Sebsbie, Anteneh Tsegaye

**Affiliations:** ^1^School of Media and Communication, Addis Ababa University, Addis Ababa, Ethiopia; ^2^Department of English Language and Literature, Kotebe University of Education, Addis Ababa, Ethiopia

**Keywords:** adolescents, entertainment screen time, screen media devices, timeframes, Uses and Gratifications Theory, media literacy

## Abstract

Adolescents' screen-based entertainment has garnered significant attention in media studies due to its multifaceted implications for academic performance, social relationships, and physical and mental wellbeing. However, the extent of their entertainment consumption and the interplay of factors affecting screen-based entertainment across devices and timeframes remain underexplored. This study investigates adolescents' Entertainment Media Screen Time (EMST) across various screen-based devices (e.g., televisions, smartphones) and different timeframes (e.g., weekdays). A survey was conducted with a stratified random sample of 720 adolescents from 56 private high schools in Addis Ababa, with participants' ages ranging from 14 to 19. Data were collected between October 18, 2024, and November 15, 2024, using a validated questionnaire (reliability Cronbach's α = 0.814). Statistical analyses comprised Spearman's Rank correlations, Chi-Square tests, Kruskal-Wallis tests with Mann-Whitney tests as *post-hoc* adjustments, applying Bonferroni correction, and the Generalized Ordinal Logistic Regression Model. Results indicated that adolescents engaged in excessive EMST [>2 h, as defined by the American Academy of Pediatrics (AAP)] across devices and timeframes at various levels. Smartphones emerged as the dominant medium for adolescents' screen-based entertainment across all timeframes, with 64.6% (on weekdays), 64.8% (on weeknights), and 81.7% (on weekend days) exceeding the 2-h threshold of EMST on these devices. Conversely, television entertainment declined, with 33.1% and 35.7% reporting no entertainment consumption during weekdays and weeknights, respectively. Significant associations were found between EMST and factors such as age, gender, grade level, parental employment, parental education, and family size (*p* < 0.05). Older adolescents were positively associated with weekday smartphone entertainment (ρ = 0.110, *p* < 0.01), while negatively correlated with television entertainment on weekend days (ρ = −0.110, *p* < 0.01). This study elucidates the complex patterns of adolescents' EMST, highlighting the roles of demographic, familial, and socioeconomic factors. Interventions should promote media literacy by raising awareness of the implications of excessive EMST engagement.

## Introduction

Adolescents' engagement with screen media has become a vital component of youth culture, meaningfully influencing their self-expression, interactions, creativity, learning, exploration, and entertainment (Kardefelt-Winther et al., 2020; Nagata et al., 2024; Schmidt-Persson et al., 2024). Nevertheless, these media are not without challenges. The American Academy of Pediatrics discourages children's and adolescents' entertainment screen time exceeding 2 h daily, emphasizing associated health implications (Council on Communications and Media et al., 2013). Other studies explain that excessive screen media use can undermine peer relationships, face-to-face interactions, family bonds, and academic performance (Beyens et al., 2020; Keles et al., 2020; Turkle, 2015; Twenge et al., 2019), highlighting the need for a broader understanding of these implications.

Existing research offers valuable insights into adolescents' screen time, with devices like smartphones increasingly recognized as the principal medium for entertainment. Their portability, versatility, and capacity to facilitate mediated social interaction have strengthened their essential role in adolescents' digital world (Huang, 2018; Twenge et al., 2019; Vorderer et al., 2016). Likewise, traditional media devices like television remain important in communal entertainment, even though their popularity is waning, mainly among older adolescents (Livingstone et al., 2018).

Differences in Entertainment Media Screen Time (EMST) by demographic factors, such as gender and age, are documented well. Older adolescents often prefer individualized screen media like smartphones for entertainment, while younger ones favor shared media platforms like televisions (Beyens et al., 2020; Livingstone and Helsper, 2007; Twenge and Campbell, 2018). Socioeconomic factors also have a role, as adolescents with more educated parents usually have greater access to media technologies (Gentile et al., 2014; Livingstone and Helsper, 2007; Nikken and Schols, 2015; Vandewater et al., 2007). Despite these findings, the interplay of adolescents' device-specific preferences, contextual factors, and temporal variations remain underexplored.

Temporal variations, which refer to differences in media consumption patterns across timeframes, such as weekdays, weeknights, and weekends illustrate the complex nature of adolescents' entertainment consumption (Livingstone et al., 2017; Shi et al., 2016; Twenge et al., 2019; Twenge and Campbell, 2018). Moreover, several prior studies fail to integrate important theoretical assumptions, constraining the broader applicability of their findings to different populations. For example, while the Uses and Gratifications Theory (Katz et al., 1974) illuminates adolescents' underlying needs and motivations for seeking entertainment, this is seldom juxtaposed with findings on EMST across timeframes, for instance, weekdays and weeknights or weekdays and weekend days. Similarly, Bronfenbrenner's (1981) Ecological Systems Theory emphasizes the role of environmental circumstances and family structures in influencing media experiences, especially in larger households (Crawford, 2020). Similarly, issues have been observed in applying the Media Affordance Theory, which explains the importance of the device's nature in affecting consumption patterns (Evans et al., 2017).

This study was conducted to address these gaps. Specifically, the objectives were to examine the extent of screen-based entertainment adolescents had across different devices (e.g., television, smartphone, tablet), and timeframes (e.g., weekday, weeknight) and to explore the factors affecting adolescents' EMST across these variables. Using descriptive and inferential analyses, the study revealed how various demographic, familial, and socioeconomic factors affect adolescents' EMST. The findings contribute to a better understanding of the dynamics of adolescents' screen media entertainment patterns, offering valuable insights that combine theoretical assumptions with empirical findings to inform policy for potential interventions.

## Methodology

### Research design

A quantitative, cross-sectional research design explored adolescents' EMST across devices (e.g., smartphones, computers, television) and timeframes (weekdays, weeknights, and weekend days). Research shows that this design is suitable for capturing trends and identifying relationships within specified timeframes (Creswell, 2003, 2008; Levin, 2006; Wang and Cheng, 2020).

### Population and sampling

The population of this study consisted of private high school adolescents in Addis Ababa. According to the Educational Statistics Annual Abstract 2021/22, published by the (Addis Ababa City Administration Education Bureau, 2023), 39, 344 private high school adolescents (grades 9–12) were enrolled in 131 schools across 11 sub-cities. The rationales for including these adolescents in this study were the following. First, research indicate that adolescents are more prone to screen-based entertainment than other age groups (Camerini et al., 2021; Council on Communications and Media, 2016; Livingstone and Helsper, 2007; Madigan et al., 2020; Priftis and Panagiotakos, 2023; Przybylski and Weinstein, 2019; Rosen et al., 2013; Throuvala et al., 2019; Twenge and Campbell, 2018; Zablotsky et al., 2024). Second, the familial socioeconomic status of these adolescents affects their ability to afford advanced screen media technologies. In other words, private high school students in Addis Ababa generally have greater access to screen-based digital devices and the internet because their families typically enjoy better financial stability compared to those from government and public schools. Most students in the latter schools rely on government-sponsored school feeding programs to attend school (Addis Ababa City Administration Education Bureau, 2023). Thus, access to these devices and the internet is relatively a luxury for these students. Third, compared to rural areas and other regions in Ethiopia, Addis Ababa, the capital city, provides stronger internet connectivity and a wider range of television coverage, thereby enhancing adolescents' access to screen-based entertainment.

For participant selection, strata were formed using the stratified random sampling technique based on the geographical locations of schools across the sub-cities to ensure proper representation. A larger sample size of 720 was selected to maintain robust statistical power across the strata—defined by the schools' locations and grade levels, while ensuring the findings' high generalizability. This was decided despite Yamane (1967) recommending a smaller sample size of 397 for a 95% confidence level and ± 5% precision, with *p* = 0.5, a trend common in other survey studies (Cochran, 1977; Fowler, 2014; Groves and Peytcheva, 2008; Lohr, 2022).

### Variables and measures

The dependent variable— EMST was measured as categorical ordinal data, representing adolescents' entertainment media screen time spent across devices (e.g., “Not at all,” “ < 1 h”). The independent variables comprised, age, gender, grade level, parental education, parental employment, and family size. A structured questionnaire was adapted from Bagot et al. (2022) and Vizcaino et al. (2019) of which content validity was confirmed through expert review. A group of experts evaluated each item for relevance, clarity, and also appropriateness to the constructs, rating them on a 4-point scale (0 = not relevant; 3 = highly relevant). The Content Validity Index was then assessed for each item and the overall scale to determine the level of agreement among experts. Additionally, qualitative feedback was gathered to identify areas needing improvement. Based on the ratings and recommendations of the experts, we revised the questionnaire to ensure alignment with the constructs. This process assured the content validity of the questionnaire, supporting its suitability for the current study. We then pilot tested it with 45 participants, which affirmed both the clarity and relevance of the instrument, yielding a reliability test result of Cronbach's alpha = 0.814, indicating acceptable internal consistency (Nunnally and Bernstein, 1994; Tavakol and Dennick, 2011; Ursachi et al., 2015).

### Data collection

Data were gathered from October 20, 2024, to November 15, 2024, through a survey questionnaire distributed in schools. Trained enumerators gave standardized directions to maintain consistency and decrease bias.

### Data analysis

Data normality was assessed using the Kolmogorov-Smirnov and Shapiro-Wilk tests, which indicated a departure from normality standards (*p*-values < 0.05). Consequently, due to the non-normal distribution of ordinal data, non-parametric tests were essential for ensuring robust analyses (Hollander et al., 2014; Howell, 2013). Percentages, medians, and interquartile ranges (IQR) were calculated as descriptive statistics. The Spearman's Rank Correlation was employed to assess rank-order associations (Akoglu, 2018; McDonald, 2014; Sheskin, 2020; Zar, 2014). The Chi-Square test was used for categorical relationships (McDonald, 2014; Sheskin, 2020), while the Kruskal-Wallis *H*-test accompanied by the Mann-Whitney *U-*test for pairwise group comparisons, with an adjusted Bonferroni correction applied (Conover and Conover, 1999; Field, 2017; McKight and Najab, 2010; Siegel and Castellan, 2003) using SPSS (Version 25). Effect Size (η^2^) of relationships was also calculated.

Additionally, the study utilized Stata 15 to run the Generalized Ordinal Logistic Regression Model (GOLRM) using the “gologit2” command along with the “autofit or” option to analyze the predictors of adolescents' EMST (Long and Freese, 2014; McCullagh, 1980). GOLRM was particularly appropriate due to observed violations of the Proportional Odds Assumption (POA), as indicated by some Likelihood Ratio (LR) tests (*p* > 0.05) and the Wald test of parallel lines assumption for the final model (*p* < 0.05; see Table 1) which are critical preconditions for using this model (Agresti, 2010; Fullerton and Xu, 2016; Liu and Agresti, 2005; Peterson and Harrell, 1990; Williams, 2006). Research indicates that the GOLRM is compatible with non-normally distributed data with ordinal outcomes (Agresti, 2010; Liu and Agresti, 2005; Williams, 2006).

**Table 1:** The Generalized Ordinal Logistic Regression Model (GOLRM) assessment on adolescents' EMST with their demographics, familial, and socioeconomic factors.

**Predictor**	**Odds Ratio (OR)**	**95% CI**	***p*-value**	**Multi-collinearity test**	**Likelihood Ratio (LR)**	**Wald test of parallel lines assumption for the final model**
**a) Weekday EMST on television-connected devices (e.g., steaming devices and game consoles)**						
Gender (Male)	0.38	(0.27, 0.53)	*p* = 0.000	VIF = 1.04	*χ^2^* = (9, *N* = 720) = 46.66 *p* = 0.000	*χ^2^* = (7, *N* = 720) = 5.36 *p* = 0.61
Mothers' unemployment	0.64	(0.44, 0.94)	*p =* 0.025	VIF = 1.13		
Fathers' unemployment	0.93	(0.42, 2.04)	*p* = 0.87	VIF = 1.03		
**b) Weekday EMST on computers/laptops**						
Fathers' education	1.31	(1.03, 1.67)	*p =* 0.024	VIF = 1.6	*χ^2^* = (9, *N* = 720) = 31.50 *p* = 0.0002	*χ^2^* = (8, *N* = 720) = 16.55 *p* = 0.035
Mothers' education	1.10	(0.87, 1.39)	*p* = 0.41	VIF = 1.7		
**c) Weekday EMST on smartphones**						
Fathers' unemployment	0.31	(0.11, 0.82)	*p* = 0.019	VIF = 1.03	*χ^2^* = (9, *N* = 720) = 30.58 *p* = 0.0003	*χ^2^* = (8, *N* = 720) = 13.60 *p* = 0.058
Mother's unemployment	0.56	(0.30, 1.03)	*p* = 0.063	VIF = 1.13		
Fathers' education	1.70	(1.19, 2.43)	*p* = 0.003	VIF = 1.6		
Mothers' education	0.83	(0.56, 1.23)	*p* = 0.36	VIF = 1.7		
**d) Weekday EMST on tablets**						
Gender (Male)	1.86	(1.34, 2.58)	*p* = 0.000	VIF = 1.04	*χ^2^* = (9, *N* = 720) = 29.99 *p* = 0.0004	*χ^2^* = (8, *N* = 720) = 11.10 *p* = 0.19
**e) Weeknight EMST on television-connected devices (e.g., steaming devices game consoles)**						
Gender (Male)	0.51	(0.36, 0.72)	*p* = 0.000	VIF = 1.04	*χ^2^* = (9, *N* = 720) = 26.53 *p* = 0.0017	*χ^2^* = (7, *N* = 720) = 4.06 *p* = 0.77
Mothers' unemployment	0.62	(0.41, 0.93)	*p* = 0.023	VIF = 1.13		
Fathers' unemployment	1.12	(0.51, 2.49)	*p* = 0.76	VIF= 1.03		
**f) Weeknight EMST on computers/laptops**						
Gender (Male)	0.69	(0.50, 0.96)	*p* = 0.029	VIF = 1.04	*χ^2^* = (9, *N* = 720) = 34.16 *p* = 0.0001	*χ^2^* = (6, *N* = 720) = 7.58 *p* = 0.27
Grade level	1.36	(1.02, 1.81)	*p* = 0.034	VIF = 3.51		
Mothers' education	1.34	(1.07, 1.67)	*p* = 0.01	VIF = 1.7		
Fathers' education	1.10	(0.87, 1.39)	*p* = 0.39	VIF = 1.6		
**g) Weeknight EMST on smartphones**						
Fathers' unemployment	0.28	(0.09, 0.84)	*p* = 0.023	VIF = 1.03	*χ^2^* = (9, *N* = 720) = 14.56 *p* = 0.103	*χ^2^* = (7, *N* = 720) = 7.48 *p* = 0.38
Mothers' unemployment	0.46	(0.24, 0.89)	*p* = 0.023	VIF = 1.13		
**h) Weeknight EMST on tablets**						
Gender (Male)	1.86	(1.33, 2.61)	*p* = 0.000	VIF = 1.04	*χ^2^* = (9, *N* = 720) = 18.88 *p* = 0.026	*χ^2^* = (8, *N* = 720) = 3.25 *p* = 0.91
**i) Weekend day EMST on television**						
Gender (Male)	1.63	(1.03, 2.57)	*p* = 0.036	VIF = 1.04	*χ^2^* = (9, *N* = 720) = 19.41 *p* = 0.022	*χ^2^* = (7, *N* = 720) = 6.66 *p* = 0.46
Family size	1.55	(1.16, 2.07)	*p* = 0.002	VIF = 1.07		
**j) Weekend day EMST on television-connected devices (e.g., streaming devices and game consoles)**						
Gender (Male)	0.37	(0.26, 0.50)	*p* = 0.000	VIF = 1.04	*χ^2^* = (9, *N* = 720) = 51.91 *p* = 0.000	*χ^2^* = (7, *N* = 720) = 6.58 *p* = 0.47
Mothers' unemployment	0.64	(0.45, 0.93)	*p* = 0.019	VIF = 1.13		
Fathers' unemployment	0.90	(0.42, 1.93)	*p* = 0.80	VIF = 1.03		
**k) Weekend day EMST on computers/laptops**						
Age	0.67	(0.51, 0.89)	*p* = 0.005	VIF = 3.64	*χ^2^* = (9, *N* = 720) = 37.60 *p* = 0.000	*χ^2^* = (5, *N* = 720) = 3.85 *p* = 0.56
Gender (Male)	0.69	(0.48, 0.99)	*p* = 0.046	VIF = 1.04		
Grade level	1.48	(1.07, 2.04)	*p* = 0.015	VIF = 3.51		
Fathers' education	1.53	(1.19, 1.96)	*p* = 0.001	VIF = 1.6		
Mothers' education	0.98	(0.77, 1.26)	*p* = 0.91	VIF = 1.7		
**l) Weekend day EMST on smartphones**						
Gender (Male)	2.72	(1.18, 6.25)	*p* = 0.018	VIF = 1.04	*χ^2^* = (9, *N* = 720) = 16.29 *p* = 0.061	*χ^2^* = (8, *N* = 720) = 9.94 *p* = 0.26
**m) Weekend day EMST on tablets**						
Gender (Male)	1.72	(1.25, 2.36)	*p* = 0.001	VIF = 1.04	*χ^2^* = (9, *N* = 720) = 26.41 *p* = 0.0017	*χ^2^* = (8, *N* = 720) = 10.89 *p* = 0.20

The table presents the Odds Ratio (OR) with their respective p-values. OR > 1 indicates higher odds of being in high EMST, while OR < 1 shows lower odds of being in high EMST. Likelihood Ratio (LR) tests and the Wald test of parallel lines assumption for the final model present the Proportional Odds Assumption (POA) for the GOLRM. Non-significant LR test results (*p* > 0.05) and significant results of the Wald tests (*p* < 0.05) signify violations of POA, proving the model's appropriateness. Variance Inflation Factor (VIF) < 10 indicates no issues of multicollinearity.

Finally, to evaluate multicollinearity, the Variance Inflation Factor (VIF) was assessed by using the “regress” and then “estat vif” commands in Stata. All VIF values were below four indicating no multicollinearity issues (see Table 1; Cohen, 1988; Field, 2017; Hair, 2010; Montgomery et al., 2020) explain that VIF values below 10 are usually acceptable.

## Results

The distribution of adolescents' EMST is summarized using descriptive statistics, such as frequency, percentage, median, and interquartile range (IQR).

As shown in Table 2, the most common EMST category for weekday television was “Not at all,” representing 33.1% of participants (*n* = 720). The median fell into this category, as well. Television entertainment spanned from no usage (33.1%) to over 4 h (1.1%). The interquartile range (IQR), capturing the middle 50% of the data, stretched from no TV entertainment to 1–2 h (21.7%), signifying that most adolescents' weekday television entertainment was under 2 h. However, a notable proportion surpassed this limit, with 11.8% of adolescents indicating 2–3 h (8.6%), 3–4 h (2.1%), and more than 4 h (1.1%).

**Table 2:** Descriptive statistics of EMST categories on weekdays across devices.

**Descriptive statistics of EMST on weekdays across devices**		**EMST categories**						
		**0 (Not at all)**	**1(**<**1 h)**	**2 (1–2 h)**	**3 (2–3 h)**	**4 (3–4 h)**	**5 (**>**4 h)**	**Total**
Television	Frequency	238	218	156	85	15	8	720
	Percentage	33.1	30.3	21.7	11.8	2.1	1.1	100.0
	Cum. percent	33.1	63.3	85.0	96.8	98.9	100.0	
	Median	1 (<1 h/weekday)						
	IQR	0 (Not at all) to 2 (1–2 h/weekday)						
Television-connected devices (eg., streaming devices, video game consoles)	Frequency	492	108	52	34	17	17	720
	Percentage	68.3	15.0	7.2	4.7	2.4	2.4	100.0
	Cum. percent	68.3	83.3	90.6	95.3	97.6	100.0	
	Median	0.00 (Not at all)						
	IQR	0 (Not at all) to 1 (< 1 h/weekday)						
Laptop/computer	Frequency	199	190	111	117	52	51	720
	Percentage	27.6	26.4	15.4	16.3	7.2	7.1	100.0
	Cum. percent	27.6	54.0	69.4	85.7	92.9	100.0	
	Median	1 (<1 h/weekday)						
	IQR	0 (Not at all) to 3 (2–3 h/weekday)						
Smartphones	Frequency	58	79	118	136	124	205	720
	Percentage	8.1	11.0	16.4	18.9	17.2	28.5	100.0
	Cum. percent	8.1	19.0	35.4	54.3	71.5	100.0	
	Median	3 (2–3 h/Weekday)						
	IQR	2 (1–2 h) to 5 (>4 h/weekday)						
Tablets	Frequency	477	93	65	42	22	21	720
	Percentage	66.3	12.9	9.0	5.8	3.1	2.9	100.0
	Cum. percent	66.3	79.2	88.2	94.0	97.1	100.0	
	Median	0.00 (Not at all)						
	IQR	0 (Not at all) to 1 (<1 h/weekday)						
Other screen media devices	Frequency	448	122	67	38	16	29	720
	Percentage	62.2	16.9	9.3	5.3	2.2	4.0	100.0
	Cum. percent	62.2	79.2	88.5	93.8	96.0	100.0	
	Median	0.00 (Not at all)						
	IQR	0 (Not at all) to 1 (<1 h/weekday)						

In addition to television entertainment, a majority of adolescents (68.3%) did not use television-connected devices (e.g., streaming devices, and game consoles) for weekday entertainment. The median also mirrored no use. The IQR revealed that the middle 50% of responses stretched from no entertainment on these devices to <1 h (15%). Only a small percentage (4.7%) went beyond the 2 h, with reported usage of these screen devices for entertainment for 2–3 h (4.7%), 3–4 h (2.4%), and more than 4 h (2.4%) on weekdays.

For laptops and computers used for entertainment, 27.6% of adolescents indicated no usage of these gadgets on weekdays. The median showed usage of <1 h (26.4%). The IQR reflected that the middle 50% of the data covered from no use to 2–3 h of EMST on these devices. Even though most adolescents reported below 2 h of entertainment, 30.6% had excessive EMST on laptops and computers: 2–3 h (16.3%), 3–4 h (7.2%), and over 4 h (7.1%).

Smartphone usage for weekday EMST was significantly higher, with only 8.1% of adolescents reporting no use. The median was 2–3 h (18.9%), and the IQR spanned from 1–2 h to over 4 h of EMST. A notable proportion of adolescents (64.6%) reported prolonged entertainment on these devices, with 2–3 h (18.9%), 3–4 h (17.2%), and more than 4 h (28.5%). These findings reflect the pervasive and protracted use of smartphones for entertainment among adolescents.

Concerning tablet use, most adolescents (66.3%) indicated no tablet-based entertainment on weekdays. The median also showed no use. The IQR ranged from no use to <1 h. Nonetheless, over 11% of adolescents had extended EMST on these devices, with reported usage of 2–3 h (5.8%), 3–4 h (3.1%), and more than 4 h (2.9%).

For “other screen media devices,” 62.2% of adolescents accounted for no use, and the median fell in this category, as well. The IQR stretched from no use to <1 h. Yet, a small proportion of adolescents had more than the 2-h entertainment limit, with 11.5% indicating EMST of 2–3 h (5.3%), 3–4 h (2.2%), and more than 4 h (4.0%).

In summary, adolescents demonstrated distinct weekday EMST patterns based on the screen device used. Limited use was observed for televisions, with 33.1% reporting no entertainment, and for television-connected devices, where 68.3% indicated no usage. Likewise, most adolescents did not use tablets for weekday entertainment (66.3%) and other screen media devices (62.2%). However, entertainment on laptops and computers was higher, with 30.6% having more than 2 h of EMST. Smartphone entertainment was the highest, with 64.6% exceeding the 2-h threshold, and 28.5% using such a device for over 4 h. These results underscore smartphones as the main contributor to extended EMST, indicating the need for interventions to promote balanced EMST among adolescents.

Similarly, this study investigated adolescents' weeknight EMST across devices.

As demonstrated in Table 3, over one-third of adolescents (35.7%) indicated no television entertainment during weeknights. The median entertainment screen time was <1 h (24%), with viewing that ranged from no use to over 4 h (4.6%). The IQR stretched from no use of the device for entertainment to 2–3 h (22.5%). Nevertheless, 28.2% of adolescents surpassed 2 h of entertainment on this screen device, with 22.5% watching for 2–3 h, 4.6% for 3–4 h, and 1.1% for more than 4 h during weeknights.

**Table 3:** Descriptive statistics of EMST categories on weeknights across devices.

**Descriptive statistics of EMST on weeknights across devices**		**EMST categories**						
		**0 (Not at all)**	**1 (**<**1 h)**	**2 (1–2 h)**	**3 (2–3 h)**	**4 (3–4 h)**	**5 (**>**4 h)**	**Total**
Television	Frequency	257	173	87	162	33	8	700
	Percentage	35.7	24.0	12.1	22.5	4.6	1.1	100.0
	Cum. percent	35.7	59.7	71.8	94.3	98.9	100.0	
	Median	1 (<1 h/weeknight)						
	IQR	0 (Not at all) to 3 (2–3 h/ weeknight)						
Television-connected devices (e.g., streaming devices, video game consoles)	Frequency	530	104	47	15	12	12	720
	Percentage	73.6	14.4	6.5	2.1	1.7	1.7	100.0
	Cum. percent	73.6	88.1	94.6	96.7	98.3	100.0	
	Median	0.00 (Not at all)						
	IQR	0 (Not at all) to 1 (<1 h/weeknight)						
Laptop/computer	Frequency	253	171	102	88	51	55	720
	Percentage	35.1	23.8	14.2	12.2	7.1	7.6	100.0
	Cum. percent	35.1	58.9	73.1	85.3	92.4	100.0	
	Median	1 (<1 h/weeknight)						
	IQR	0 (Not at all) to 3 (2–3 h/weeknight)						
Smartphones	Frequency	47	99	107	155	116	196	720
	Percentage	6.5	13.8	14.9	21.5	16.1	27.2	100.0
	Cum. percent	6.5	20.3	35.1	56.7	72.8	100.0	
	Median	3 (2–3 h/weeknight)						
	IQR	2 (1–2 h) to 5 (>4 h/weeknight)						
Tablets	Frequency	505	89	42	39	26	19	720
	Percentage	70.1	12.4	5.8	5.4	3.6	2.6	100.0
	Cum. percent	70.1	82.5	88.3	93.8	97.4	100.0	
	Median	0.00 (Not at all)						
	IQR	0 (Not at all) to 1 (<1 h/weeknight)						
Other screen media devices	Frequency	503	95	54	32	13	23	720
	Percentage	69.9	13.2	7.5	4.4	1.8	3.2	100.0
	Cum. percent	69.9	83.1	90.6	95.0	96.8	100.0	
	Median	0.00 (Not at all)						
	IQR	0 (Not at all) to 1 (<1 h/weeknight)						

Apart from television usage, a majority (73.6%) reported no screen-based entertainment on television-connected devices (e.g., streaming devices, and game consoles) during weeknights. Usage spanned from no use (median) to over 4 h (1.7%). The IQR suggested minimal use, from no entertainment on these devices to <1 h. Only 5.5% had extended EMST, with 2.1% using it for 2–3 h and smaller proportions spending for 3–4 h (1.7%), and over 4 h (1.7%).

Regarding laptops and computer-based entertainment, more than one-third (35.1%) reported no consumption, and the median was below 1 h (23.8%). Entertainment on these devices ranged widely, with 7.6% consuming for over 4 h. The IQR spanned from no use to 2–3 h, and 26.9% reported lengthy entertainment on these devices, with 12.2% having EMST for 2–3 h, 7.1% for 3–4 h, and 7.6% for more than 4 h.

Similarly, weeknight smartphone entertainment was notably high, with only 6.5% of adolescents reporting no use. The median was 2–3 h (21.5%), and 27.2% indicated having EMST that exceeded 4 h. The IQR covered from 1–2 h to over 4 h. A large proportion of adolescents (64.8%) had excessive EMST on these devices, with 21.5% for 2–3 h, 16.1% for 3–4 h, and 27.2% for more than 4 h.

Concerning tablet use for entertainment, most adolescents (70.1%) reported no tablet-based entertainment during weeknights, with the median also reflecting no use. EMST on these devices stretched from none to over 4 h (2.6%). The IQR extended from no entertainment on these devices to <1 h. Excessive EMST was notable in 11.6% of participants, including 5.4% using them for 2–3 h, 3.6% for 3–4 h, and 2.6% for over 4 h.

What's more, for “other screen media devices,” a remarkable majority (69.9%) did not use such screens for entertainment during weeknights. Entertainment on these devices spanned from none (median) to more than 4 h (3.2%). The IQR ranged from no use to <1 h. Only 9.4% had extended entertainment, with 4.4% reporting the use of these devices for 2–3 h, 1.8% for 3–4 h, and 3.2% for over 4 h.

In summary, weeknight EMST differed by device. Television and television-connected devices reflected limited use, with 35.7% and 73.6% of adolescents, respectively, reporting no entertainment on these devices. Laptops and computer-based EMST were higher, with 26.9% exceeding 2 h. Smartphone entertainment stood out, as 64.8% reported consumption on these devices higher than the 2-h limit, including 27.2% consuming for more than 4 h. Entertainment on tablets and other screen media devices was less common, with over 70% reporting no entertainment on these gadgets, while around 10% exceeded the limit. Smartphones remain the principal driver of prolonged EMST during weeknights.

Similarly, this study investigated adolescents' weekend days' EMST across devices.

As depicted in Table 4, about 25.1% of adolescents spent 2–3 h watching television entertainment during weekend days, the highest reported consumption, while 12.6% reported no television-based entertainment. The median entertainment screen time fell 2–3 h, with an IQR covering from no use to 2–3 h. Extended television entertainment was substantial, with 25.1%, 18.1%, and 11% of adolescents viewing for 2–3 h, 3–4 h, and more than 4 h, respectively.

**Table 4:** Descriptive statistics for EMST categories on weekend day.

**Descriptive statistics of EMST on weekend days across devices**		**EMST categories**						
		**0 (Not at all)**	**1 (**<**1 h)**	**2 (1–2 h)**	**3 (2–3 h)**	**4 (3–4 h)**	**5 (**>**4 h)**	**Total**
Television	Frequency	91	113	126	181	130	79	720
	Percentage	12.6	15.7	17.5	25.1	18.1	11.0	100.0
	Cum. percent	12.6	28.3	45.8	71.0	89.0	100.0	
	Median	3 (2–3 h/ weekend day)						
	IQR	0 (Not at all) to 3 (2–3 h/weekend day)						
Television-connected devices (e.g., streaming devices, video game console)	Frequency	433	124	68	43	26	26	720
	Percentage	60.1	17.2	9.4	6.0	3.6	3.6	100.0
	Cum. percent	60.1	77.4	86.8	92.8	96.4	100.0	
	Mode	0 (Not at all)						
	Median	0.00 (Not at all)						
	IQR	0 (Not at all) to 1 (<1 h/weekend day)						
Laptop/computer	Frequency	175	135	115	103	88	104	720
	Percentage	24.3	18.8	16.0	14.3	12.2	14.4	100.0
	Cum. percent	24.3	43.1	59.0	73.3	85.6	100.0	
	Median	2 (1–2 h/weekend day)						
	IQR	0 (Not at all) to 3 (2–3 h/weekend day)						
Smartphones	Frequency	29	47	56	110	144	334	720
	Percentage	4.0	6.5	7.8	15.3	20.0	46.4	100.0
	Cum. percent	4.0	10.6	18.3	33.6	53.6	100.0	
	Median	4 (3–4 h/ weekend day)						
	IQR	2 (1–2 h) to 5 (>4 h/weekend day)						
Tablets	Frequency	451	87	46	48	45	43	720
	Percentage	62.6	12.1	6.4	6.7	6.3	6.0	100.0
	Cum. percent	62.6	74.7	81.1	87.8	94.0	100.0	
	Median	0.00 (Not at all)						
	IQR	0 (Not at all) to 1 (<1 h/weekend day)						
Other screen media devices	Frequency	448	110	63	30	28	41	720
	Percentage	62.2	15.3	8.8	4.2	3.9	5.7	100.0
	Cum. percent	62.2	77.5	86.3	90.4	94.3	100.0	
	Median	0.00 (Not at all)						
	IQR	0 (Not at all) to 1 (<1 h/weekend day)						

On the other hand, most adolescents (60.1%) reported no entertainment on television-connected devices (e.g., streaming devices or game consoles) during weekend days. Nevertheless, 3.6% indicated consumption that exceeded 4 h. The median entertainment screen time on such devices was “not at all,” and the IQR spanned from no use to <1 h. Excessive entertainment screen time was relatively less, with 6.0%, 3.6%, and 3.6% of adolescents indicating 2–3 h, 3–4 h, and over 4 h of entertainment, respectively.

Likewise, while 24.3% of adolescents indicated no entertainment on laptops and computers, 14.4% spent over 4 h on these devices on a weekend day. The median entertainment screen time was 1–2 h, with an IQR stretching from no use to 2–3 h of entertainment. Excessive EMST was significant, with 14.3%, 12.2%, and 14.4% using laptops and computer-based entertainment for 2–3 h, 3–4 h, and more than 4 h, respectively.

Similarly, smartphone entertainment was the most consumed, with only 4.0% of adolescents reporting no entertainment, while 46.4% surpassed 4 h during weekend days. This device's median entertainment screen time was 3–4 h, and the IQR stretched from 1–2 h to over 4 h. Excessive entertainment EMST on smartphones was widespread, with 15.3%, 20%, and 46.4% indicating 2–3 h, 3–4 h, and more than 4 h, respectively.

Regarding tablet use for entertainment, most adolescents (62.6%) reported no tablet-based entertainment, while 6.0% spent more than 4 h on weekend days. The median entertainment on this device was “not at all,” with an IQR covering from no use to <1 h. Extended tablet entertainment was noted in 6.7%, 6.3%, and 6.0% of adolescents, who consumed these devices for 2–3 h, 3–4 h, and more than 4 h, respectively.

In addition, 62.2% of adolescents reported no use of other screen media devices for entertainment, although 5.7% reported over 4 h of entertainment. The median was “not at all,” with an IQR extending from no use to <1 h. Excessive EMST on other screen media devices was shown among 4.2%, 3.9%, and 5.7% of adolescents, with 2–3 h, 3–4 h, and more than 4 h, respectively.

In sum, adolescents' weekend days' EMST fluctuated by devices. Television-based entertainment was notable, with 25.1% viewing over 4 h, whereas 12.6% reported no entertainment. Entertainment consumption on television-connected gadgets was small, as 60.1% reported no use of these devices, and only 3.6% surpassed 4 h. Laptops and computer-based entertainment were relatively high, with 14.4% consuming more than 4 h. Smartphone entertainment was the leading entertainment among participants, with 46.4% exceeding 4 h, while only 4.0% reported no entertainment on such screen devices. Tablets and other screen media devices were relatively less consumed for entertainment, with over 60% indicating no entertainment on these devices. However, about 6% of adolescents spent more than 4 h of entertainment using these devices. In general, smartphone entertainment emerged as the principal source of excessive EMST during weekend days.

### Age and EMST

Table 5 demonstrates the relationship between adolescents' age and EMST across various devices, providing valuable insights into changing media consumption patterns. Spearman's Rank Correlation revealed a weak but significant positive relationship between age and weekday smartphone use for entertainment (ρ = 0.110, *p* < 0.01), indicating that older adolescents spend more time on smartphone entertainment during weekdays, with a small effect size (η^2^ = 0.012). This increase may be due to greater autonomy or social pressures, suggesting the need for interventions to manage excessive entertainment among older adolescents.

**Table 5:** Spearman's rank correlations between socio-demographics characteristics and EMST with their effect size (η^2^).

**Correlation**	**Device**	**Time**	**Test statistics**	***p*-value**	**Effect size (*η^2^*)**
Age and EMST	Smartphone	Weekday	ρ = 0.110^**^	*p* < 0.01	0.012
	Tablet	Weekday	ρ = −0.129^**^	*p* < 0.01	0.016
	Tablet	Weeknight	ρ = −0.084^*^	*p* < 0.05	0.007
	Television	Weekend days	ρ = −0.110^**^	*p* < 0.01	0.012
	Tablet	Weekend days	ρ = −0.130^**^	*p* < 0.01	0.016
	Other screen media devices	Weekend days	ρ = −0.098^**^	*p* < 0.01	0.009
Grade levels and EMST	Smartphone	Weekday	ρ = 0.091^*^	*p* < 0.05	0.008
	Smartphone	Weeknight	ρ = 0.080^*^	*p* < 0.05	0.006
	Smartphone	Weekend days	ρ = 0.084^*^	*p* < 0.05	0.007
	Tablet	Weekday	ρ = −0.097^**^	*p* < 0.01	0.009
	Tablet	Weekend days	ρ = −0.112^**^	*p* < 0.01	0.012
	Television	Weekend days	ρ = −0.084^*^	*p* < 0.05	0.007
	Other screen media devices	Weekend days	ρ = −0.108^**^	*p* < 0.01	0.011
Mothers' education and children's EMST	Laptops/computers	Weekday	ρ = 0.141^**^	*p* < 0.01	0.019
	Laptops/computers	Weeknight	ρ = 0.139^**^	*p* < 0.01	0.019
	Laptops/computers	Weekend days	ρ = 0.150^**^	*p* < 0.01	0.022
	Smartphones	Weeknight	ρ = −0.044	*p* > 0.05	0.001
	Tablets	Weekend days	ρ = 0.025	*p* > 0.05	0.000
Fathers' education and children's EMST	Laptops/computers	Weekday	ρ = 0.112^**^	*p* < 0.01	0.012
	Laptops/computers	Weeknight	ρ = 0.139^**^	*p* < 0.01	0.019
	Laptops/computers	Weekend days	ρ = 0.159^**^	*p* < 0.01	0.025
	Television	Weekday	ρ = −0.073^*^	*p* < 0.05	0.005
	Smartphones	Weekday	ρ = 0.005	*p* < 0.05	0.000
Family size and adolescents' EMST	Smartphones	Weekday	ρ = −0.008	*p* > 0.05	0.000
	Television	Weeknight	ρ = 0.058	*p* > 0.05	0.003
	Laptops/computers	Weekend days	ρ = −0.027	*p* > 0.05	0.000
	Television	Weekend days	ρ = 0.098^**^	*p* < 0.01	0.009

One asterisk (^*^) indicates that the Spearman's Rank correlation is significant at the 0.05 level (*p* < 0.05).

Two asterisks (^**^) show statistical significance at the 0.01 level (*p* < 0.01). Effect size (η^2^) < 0.01 is negligible; 0.01 ≤ η^2^ < 0.06 is small; 0.06 ≤ η^2^ < 0.14 is medium; η^2^ ≥ 0.14 is large (Cohen, 1988).

Conversely, a weak negative correlation was found between age and weekday EMST on tablets (ρ = −0.129, *p* < 0.01), indicating a decline in tablet usage as adolescents age. A similar negative correlation was observed for weeknight entertainment on tablets (ρ = −0.084, *p* < 0.05), with older adolescents spending less time on these devices, possibly due to busier academic schedules or the appeal of more versatile devices. The effect sizes for both correlations were small (η^2^ = 0.016 and 0.007, respectively).

On weekend days, older adolescents were less likely to use televisions for entertainment, as evidenced by a weak negative correlation (ρ = −0.110, *p* < 0.01). Similarly, weekend day tablet usage for entertainment decreased with age (ρ = −0.130, *p* < 0.01), reinforcing the shift toward more personalized, portable devices, with small effect sizes (η^2^ = 0.012 and 0.016, respectively).

A particularly strong, yet negative association was observed between age and the use of other screen media devices for entertainment during weekend days (ρ = −0.98, *p* < 0.01), reflecting a small effect size (η^2^ = 0.009). This sharp decline highlights that older adolescents are increasingly moving away from other screen media devices toward smartphones, which offer a more portable and integrated entertainment experience.

In summary, as adolescent's age, they increasingly favor smartphones for entertainment, especially on weekdays, while tablet, television, and other screen media device usage declines. The effect size in all associations was small. These trends underscore the importance of age-specific interventions to manage excessive smartphone use for entertainment among older adolescents while promoting balanced screen time for younger adolescents who still rely more on tablets and other screen media devices.

### Grade level and EMST

Spearman's Rank Correlation also explored the relationship between adolescents' grade levels and entertainment screen time across different devices. The analysis revealed a weak but statistically significant positive correlation between grade levels and smartphone entertainment usage on weekdays (ρ = 0.091, *p* < 0.05), weeknights (ρ = 0.080, *p* < 0.05), and weekend days (ρ = 0.084, *p* < 0.05). This suggests that as students' progress through grade levels, their use of smartphones for entertainment increases, albeit with small effect sizes (η^2^ = 0.008; 0.006; 0.007, respectively).

Conversely, a weak but statistically significant negative correlation was found between grade levels and tablet-based entertainment on weekdays (ρ = −0.097, *p* < 0.01) and weekend days (ρ = −0.112, *p* < 0.01), indicating a decline in tablet use for entertainment as students advance in grade levels. The effect sizes for these associations were also small (η^2^ = 0.009 and 0.012, respectively). A similar trend was observed in the associations between grade level and students' television and other screen media entertainment on weekend days (ρ = −0.084, *p* < 0.05; ρ = −0.108, *p* < 0.01), suggesting that students may be shifting away from weekend day entertainment on these devices in favor of more versatile options, such as smartphones, or due to other responsibilities. The effect sizes in both cases were small (η^2^ = 0.007 and 0.011, respectively).

In summary, students' smartphone entertainment patterns show a positive association with grade levels across all time categories, while entertainment through tablets, television, and other screen media decreases as grade levels rise, particularly on weekend days. This highlights the need for targeted interventions to promote balanced entertainment consumption, despite the consistently small effect sizes.

### Parental education and EMST

The analysis of adolescents' EMST and parental education revealed a weak yet statistically significant positive correlation between mothers' high educational attainment and children's entertainment screen time on laptops and computers during weekdays, weeknights, and weekend days (ρ = 0.141, *p* < 0.01; ρ = 0.139, *p* < 0.01; ρ = 0.150, *p* < 0.01, respectively), indicating small effect sizes in all associations (η^2^ = 0.019; 0.019; 0.022, respectively). A parallel analysis found a significant positive association between fathers' education and children's entertainment screen time on laptops or computers across the same periods, with similar results for children's weeknight entertainment (ρ = 0.112, *p* < 0.01; ρ = 0.139, *p* < 0.01; ρ = 0.159, *p* < 0.01). These findings suggest that more educated parents are more likely to provide such resources to their children, leading to increased EMST. The effect sizes for all were small (η^2^ = 0.012; 0.029; 0.025, respectively).

The analysis also revealed a weak negative but significant association between fathers' education and children's television entertainment (ρ = −0.073, *p* < 0.05), with a small effect size (η^2^ = 0.005). This indicates that children with less-educated fathers relied more on television for entertainment, while those with better-educated fathers spent more time on tablet-based entertainment. However, no significant relationship was observed between parental education and adolescents' entertainment screen time on other devices. For example, there was no association between fathers' education and children's weekday smartphone use for entertainment (ρ = 0.005, *p* > 0.05), nor between mothers' education and children's weeknight smartphone use (ρ = −0.044, *p* > 0.05) and weekend day tablet-based entertainment (ρ = 0.025, *p* > 0.05). This lack of association may suggest that other contextual factors influence adolescents' entertainment choices. Overall, these findings highlight the influence of parental education on children's EMST, suggesting interventions that promote balanced entertainment screen time.

### Family size and EMST

Family size and adolescents' EMST showed no statistically significant association in most cases, including family size with adolescents' weekday smartphone entertainment, weeknight television entertainment, and weekend day laptop/computer-based entertainment (ρ = −0.008, *p* > 0.05; ρ = 0.058, *p* > 0.05; and ρ = −0.027, *p* > 0.05, respectively). This indicates that family size does not impact adolescents' EMST across these devices and timeframes. In contrast, a weak yet highly significant positive association was found between family size and adolescents' weekend day television entertainment (ρ = 0.098, *p* < 0.01), with a small effect size (η^2^ = 0.009). This suggests that weekend day television entertainment increases with family size, implying that larger families may find it more challenging to monitor their children's television-based entertainment.

The consistently small effect sizes across all relationships indicate that while the associations are statistically significant, they have minimal practical significance. This highlights the role of these socio-demographic factors in influencing adolescents' entertainment across various screen-based digital devices and timeframes.

As displayed in Table 6, a Chi-Square test of independence identified a significant relationship between gender and entertainment screen time on weekend days using televisions, χ^2^ (5, *N* = 720) = 16.153, *p* = 0.006. *Post-hoc* analysis revealed that female adolescents were significantly over-represented in the >4 h category (SR = 2.1), whereas male adolescents were under-represented (SR = −2.3), indicating that females are more likely to engage in prolonged television viewing on weekends.

**Table 6:** A Chi-Square test of independence and SR as *post-hoc* test of gender and EMST.

**Chi-Square test**	**Gender**	**EMS category**	**Observed count**		**Expected count**		***Post-hoc*** **(Standardized Residual) SR**	
*χ^2^* (5, *N* = 720) = 16.153, *p* = 0.006 (Gender and weekend day entertainment on television)	Male	2–3 h	91		84.2		0.7	
		3–4 h	58		60.5		−0.3	
		>4 h	23		36.8		−2.3^*^	
	Female	2–3 h	90		96.8		−0.7	
		3–4 h	72		69.5		0.3	
		>4 h	56		42.2		2.1^*^	
*χ^2^* (5, *N* = 720) = 44.117, *p* = 0.000; *χ^2^* (5, *N* = 720) = 71.097, *p* = 0.000 (Gender and weekday; weekend day entertainment on TV-connected devices, respectively)	Male	2–3 h	19	34	15.8	20	0.8	3.1^*^
		3–4 h	14	15	7.9	12.1	2.2^*^	0.8
		>4 h	15	24	7.9	12.1	2.5^*^	3.4^*^
	Female	2–3 h	15	9	18.2	23	−0.7	−2.9^*^
		3–4 h	3	11	9.1	13.9	−2.0^*^	−0.8
		>4 h	2	2	9.1	13.9	−2.4^*^	−3.2^*^
*χ^2^* (5, *N* = 720) = 20.025, *p* = 0.001 (Gender and weekday entertainment on laptops/ computers)	Male	2–3 h	59		54.4		0.6	
		3–4 h	24		24.2		0.0	
		>4 h	38		23.7		2.9^*^	
	Female	2–3 h	58		62.6		−0.6	
		3–4 h	28		27.8		0.0	
		>4 h	13		27.3		−2.7^*^	
*χ^2^* (5, *N* = 720) = 13.942, *p* = 0.016; *χ^2^* (5, *N* = 720) = 11.478, *p* = 0.043 (Gender and weeknight; weekend day entertainment on smartphones, respectively)	Male	2–3 h	77	53	72.1	51.2	0.6	0.3
		3–4 h	50	65	54.0	67	−0.5	−0.2
		>4 h	73	143	91.2	155.4	– 1.9	−1.0
	Female	2–3 h	78	57	82.9	58.8	−0.5	−0.2
		3–4 h	66	79	62.0	77	0.5	0.2
		>4 h	123	191	104.8	178.6	1.8	0.9
*χ^2^* (5, *N* = 720) = 21.874, *p* = 0.001; *χ^2^* (5, *N* = 720) = 19.845, *p* = 0.001 (Gender and weekday; weeknight entertainment on tablets, respectively)	Male	2–3 h	10	13	19.5	18.1	−2.2^*^	−1.2
		3–4 h	7	8	10.2	12.1	−1.0	−1.2
		>4 h	7	3	9.8	8.8	−0.9	−2.0^*^
	Female	2–3 h	32	26	22.5	20.9	2.0^*^	1.1
		3–4 h	15	18	11.8	13.9	0.9	1.1
		>4 h	14	16	11.2	10.2	0.8	1.8
*χ^2^* (5, *N* = 720) = 15.236, *p* = 0.009 (Gender and weekday entertainment on other screen media devices)	Male	2–3 h	10		17.7		−1.8	
		3–4 h	4		7.4		−1.3	
		>4 h	10		13.5		−1.0	
	Female	2–3 h	28		20.3		1.7	
		3–4 h	12		8.6		1.2	
		>4 h	19		15.5		0.9	

The table presents excessive EMST categories (>2 h/day as defined by the American Academy of Pediatrics, Council on Communications and Media et al., 2013). *Post-hoc* tests using Standardized Residuals (SR) > +1.96 or < -1.96 are marked with an asterisk (^*^), indicating statistically significant over-or under-representation of EMST for their respective category.

Similarly, Chi-Square tests for weekday and weekend day entertainment screen time using television-connected devices (e.g., streaming devices and game consoles) were significant: χ^2^ (5, *N* = 720) = 44.177, *p* < 0.001 (weekdays), and χ^2^ (5, *N* = 720) = 71.097, *p* < 0.001 (weekend days). *Post-hoc* analysis showed that male adolescents were over-represented in the 3–4 h (SR = 2.2) and >4 h (SR = 2.5) categories on weekdays, with female under-representation (SR = −2.0 and −2.4). On weekend days, males were over-represented in the 2–3 h (SR = 3.1) and >4 h (SR = 3.4) categories, while females were under-represented (SR = −2.9 and −3.2), suggesting a higher likelihood of males using these devices for entertainment.

A Chi-Square test for weekday entertainment screen time using laptops and computers was significant, χ^2^ (5, *N* = 720) = 20.025, *p* = 0.001. Males were overrepresented in the >4 h category (SR = 2.9), and females were underrepresented (SR = −2.7), indicating that males spent more time on these devices for entertainment.

While gender was significantly associated with smartphone entertainment on weekdays and weekend days, χ^2^ (5, *N* = 720) = 13.942, *p* = 0.016, and χ^2^ (5, *N* = 720) = 11.478, *p* = 0.043, *post-hoc* analysis showed no significant gender differences, as standardized residuals were not significant.

For weekday and weekend day tablet-based entertainment, Chi-Square tests showed a significant correlation, χ^2^ (5, *N* = 720) = 21.874, *p* = 0.001, and χ^2^ (5, *N* = 720) = 19.845, *p* = 0.001, respectively. Male adolescents were under-represented in the 2–3 h category (SR = −2.2), while their female counterparts were over-represented (SR = 2.0). Males were under-represented (SR = −2.0) in the >4 h category, while females came close to over-representation (SR = 1.8), emphasizing gender differences in tablet-based entertainment.

A Chi-Square test for weekday entertainment on other screen media devices was significant, χ^2^ (5, *N* = 720) = 15.236, *p* = 0.009 although *post-hoc* analysis revealed no significant gender disparity in the EMST category. This suggests no variation between genders in their entertainment consumption on other screen media devices.

Overall, the analyses revealed important patterns in the relationship between gender and EMST. Over-representation of male adolescents in higher EMST categories for laptops and television-connected devices was common, especially during weekdays, signifying a higher likelihood of entertainment on these devices. Females, on the other hand, preferred television and tablet-based entertainment on weekend days. Despite these variations, there was no significant gender difference in using smartphones and other screen media devices for entertainment, suggesting the alignment of certain screen devices with gender trends for entertainment, while others are more or less equally popular for both sexes.

As illustrated in Table 7, a series of Chi-Square tests revealed the influence of parental employment status on adolescents' EMST across various devices and timeframes. For mothers' employment, significant associations were identified for both weekday entertainment, χ^2^ (5, *N* = 720) = 12.941, *p* = 0.024, and weeknight entertainment, χ^2^ (5, *N* = 720) = 15.056, *p* = 0.010, on television-connected devices, such as streaming devices and video game consoles. *Post-hoc* analyses indicated no significant variations in most EMST categories, as standardized residuals (SR) showed any over- or under-representation. Nevertheless, a notable under-representation was observed in the 3–4 h EMST category for adolescents with unemployed mothers on weekdays (SR = −2.3) and weeknights (SR = −2.0). This suggests that adolescents with unemployed mothers engaged less in entertainment content consumption on these devices during these periods. A similar trend was noted for mothers' employment and adolescents' weeknight smartphone entertainment. The Chi-Square test showed a significant relationship, χ^2^ (5, *N* = 720) = 11.075, *p* = 0.050, with most EMST categories revealing no significant deviation. However, there was a significant under-representation of adolescents with unemployed mothers (SR = −2.1) in the 2–3 h EMST category, indicating reduced smartphone entertainment on weeknights.

**Table 7:** A Chi-Square test of independence and standardized residual as *post-hoc* test of parental employment status and adolescents' EMST.

**Chi-Square test**	**Employed**	**EMST category**	**Observed count**	**Expected count**	***Post-hoc* (Standardized Residual) SR**
*χ^2^* (5, *N* = 720) = 12.941, *p* = 0.024 (Mothers' employment and adolescents' weekday entertainment on television-connected devices e.g., streaming devices, videogame consoles	Yes	2–3 h	26	23.1	0.6
		3–4 h	17	11.6	1.6
		>4 h	11	11.6	−0.2
	No	2–3 h	8	10.9	−0.9
		3–4 h	0	5.4	−2.3^*^
		>4 h	6	5.4	0.2
*χ^2^* (5, *N* = 720) = 15.056, *p* = 0.010 (Mothers' employment and adolescents' weeknight entertainment on television-connected devices e.g., streaming devices, videogame consoles)	Yes	2–3 h	14	10.2	1.2
		3–4 h	12	8.2	1.3
		>4 h	10	8.2	0.6
	No	2–3 h	1	4.8	−1.7
		3–4 h	0	3.8	−2.0^*^
		>4 h	2	3.8	−0.9
*χ^2^* (5, *N* = 720) = 11.075, *p* = 0.050 (Mothers' employment and adolescents' weeknight entertainment on smartphones)	Yes	2–3 h	120	105.5	1.4
		3–4 h	74	78.9	−0.6
		>4 h	136	133.4	0.2
	No	2–3 h	35	49.5	−2.1^*^
		3–4 h	42	37.1	0.8
		>4 h	60	62.5	−0.3
*χ^2^* (5, *N* = 720) = 11.787, *p* = 0.038 (Fathers' employment and adolescents' weekday entertainment on smartphones)	Yes	2–3 h	134	130	0.4
		3–4 h	117	118.5	−0.1
		>4 h	198	195.9	0.2
	No	2–3 h	2	6.0	−1.6
		3–4 h	7	5.5	0.6
		>4 h	7	9.1	−0.7
*χ^2^* (5, *N* = 720) = 12.616, *p* = 0.027 (Fathers' employment and adolescents' weeknight entertainment on smartphones)	Yes	2–3 h	145	148.1	−0.3
		3–4 h	116	110.8	0.5
		>4 h	190	187.3	0.2
	No	2–3 h	10	6.9	1.2
		3–4 h	0	5.2	−2.3^*^
		>4 h	6	8.7	−0.9
*χ^2^* (5, *N* = 720) = 15.482, *p* = 0.008 (Fathers' employment and adolescents' weekend day entertainment on smartphones)	Yes	2–3 h	107	105.1	0.2
		3–4 h	135	137.6	−0.2
		>4 h	327	319.2	0.4
	No	2–3 h	3	4.9	−0.9
		3–4 h	9	6.4	1.0
		>4 h	7	14.8	−2.0^*^

The table presents excessive EMST categories (>2 h/day). *Post-hoc* tests using Standardized Residuals (SR) > +1.96 or < -1.96 are marked with an asterisk (^*^), indicating statistically significant over-or under-representation of EMST for the respective category.

Regarding fathers' employment and adolescents' entertainment, significant relationships were observed for adolescents' smartphone entertainment during weekdays, weeknights, and weekend days. For weekday entertainment, χ^2^ (5, *N* = 720) = 11.787, *p* = 0.038, the *post-hoc* analysis indicated no significant variations across all categories of EMST. However, for weeknight, χ^2^ (5, *N* = 720) = 12.616, *p* = 0.027, and weekend day entertainment, χ^2^ (5, *N* = 720) = 15.482, *p* = 0.008, there was notable underrepresentation in the 2–3 h (SR = −2.3), and >4 h (SR = −2.0) EMST categories, respectively. In other terms, paternal unemployment was associated with decreased adolescents' entertainment on smartphones during weeknights and weekend days.

In summary, the analysis demonstrated that adolescents with unemployed parents were less likely to spend prolonged EMST. The under-representation predominantly occurred in the 2–4 h EMST category during weekdays and weeknights. Adolescents who had unemployed parents spent less EMST on television-connected devices and smartphones.

As depicted in Table 8A, the Kruskal-Wallis *H*-test was employed to examine adolescents' EMST across various devices and grade levels, followed by *post-hoc* pairwise comparisons (Table 8B) using the Mann-Whitney *U*-test, adjusted with Bonferroni correction (*p* < 0.0083). Mean Rank (MR) and effect sizes (η^2^) were also calculated for the *post-hoc* comparisons.

**Table 8A:** Kruskal-Wallis *H-*test on the adolescents' grade levels and EMST.

**Test**	**Time**	**Screen media device**	**H-statistics**	***p*-value**
Kruskal-Wallis *H-*test	Weekdays	Television	*H* = 11.872	*p* = 0.008
	Weekdays	Laptops/computers	*H* = 9.906	*p* = 0.019
	Weekdays	Smartphones	*H* = 10.679	*p* = 0.014
	Weekdays	Tablets	*H* = 8.946	*p* = 0.030
	Weeknights	TV-connected devices	*H* = 8.955	*p* = 0.030
	Weeknights	Laptops/computers	*H* = 11.355	*p* = 0.010
	Weeknights	Smartphones	*H* = 12.509	*p* = 0.006
	Weekend days	Television	*H* = 10.325	*p* = 0.016
	Weekend days	TV-connected devices	*H* = 9.302	*p* = 0.026
	Weekend days	Smartphones	*H* = 11.377	*p* = 0.010
	Weekend days	Tablets	*H* = 9.294	*p* = 0.026
	Weekend days	Other screen media devices	*H* = 9.881	*p* = 0.020

Statistical significance for a Kruskal-Wallis H-test is *p* < 0.05.

**Table 8B:** *Post-hoc* pairwise comparisons using Mann-Whitney *U-*test with Bonferroni correction (adjusted *p* < 0.0083), mean rank, and effect size (η^2^) between adolescents' grade levels and EMST.

**Comparison between grade levels**	**Time**	**Screen media device**	**U-statistics**	**Mann-Whitney *U*-test *p*-value**	**Mean ranks of grade levels on EMST**	**Effect size (*η^2^*)**
Grade 9 vs. Grade 10	Weekdays	Laptop/computer	*U* = 9,461	*p* = 0.002	Grade 9 = 141.80 Grade 10 = 175.10	0.029
	Weeknights	Laptop/computer	*U* = 9,528	*p* = 0.003	Grade 9 = 142.41 Grade 10 = 174.79	0.027
	Weekend days	Televisions	*U* = 9,688.5	*p* = 0.005	Grade 9 = 184.11 Grade 10 = 153.94	0.023
	Weekend days	Smartphones	*U* = 9,766.5	*p* = 0.006	Grade 9= 144.60 Grade 10 = 173.70	0.023
Grade 9 vs. Grade 11	Weekdays	Smartphones	*U* = 9,013	*p* = 0.007	Grade 9 = 137.69 Grade 11 = 165.88	0.023
	Weeknights	Smartphones	*U* = 8,409	*p* = 0.000	Grade 9 = 132.15 Grade 11 = 168.89	0.039
	Weekend days	Smartphones	*U* = 8,730	*p* = 0.002	Grade 9 = 135.09 Grade 11 = 167.28	0.032
	Weekdays	Televisions	*U* = 8,555.5	*p* = 0.001	Grade 9 = 178.51 Grade 11 = 143.85	0.036
Grade 9 vs. Grade 12	Weeknights	Laptop/computer	*U* = 8,546	*p* = 0.007	Grade 9 = 133.40 Grade 12 = 160.26	0.024
	Weekend days	Smartphones	*U* = 8,486	*p* = 0.005	Grade 9 = 132.85 Grade 12 = 160.57	0.026
		Televisions	*U* = 8,292	*p* = 0.003	Grade 9 = 169.93 Grade 12=139.41	0.029
		Tablets	*U* = 8,684.5	*p* = 0.005	Grade 9 = 166.33 Grade 12 = 141.47	0.026
		Other screen media devices	*U* = 8,799	*p* = 0.008	Grade 9 = 165.28 Grade 12 = 142.07	0.023
Grade 10 vs. Grade 11	Weekdays	Smartphones	*U* = 18,787	*p* = 0.007	Grade 10 = 195.50 Grade 11 = 226.69	0.017
Grade 10 vs. Grade 12	Weekdays	Tablets	*U* = 17,983.5	*p* = 0.004	Grade 10 = 218.01 Grade 12 = 190.15	0.020
	Weeknights	Tablets	*U* = 18,312	*p* = 0.008	Grade 10 = 216.50 Grade 12 = 191.87	0.016
	Weekend days	Other screen media devices	*U* = 17,996	*p* = 0.006	Grade 10 = 217.95 Grade 12 = 190.22	0.018
Grade 11 vs. Grade 12	Weeknights	Television-connected devices	*U* = 16,730.5	*p* = 0.004	Grade 11 = 209.68 Grade 12 = 183.59	0.021
	Weekend days	Television-connected devices	*U* = 16,426	*p* = 0.004	Grade 11 = 211.18 Grade 12 = 182.00	0.021

Statistical significance of a *post-hoc* Mann-Whitney pairwise comparison with Bonferroni correction (adjusted p < 0.0083). Effect size (η^2^) < 0.01 is negligible; 0.01 ≤ η^2^ < 0.06 is small; 0.06 ≤ η^2^ < 0.14 is medium; η^2^ ≥ 0.14 is large (Cohen, 1988).

The findings of the Kruskal-Wallis *H*-test indicated statistically significant variations in EMST across grade levels. Specifically, significant differences were observed in weekday [H (3) = 9.906, *p* = 0.019] and weeknight [H (3) = 11.355, *p* = 0.010] laptop and computer use, as well as weekend day television use for entertainment [H (3) = 10.325, *p* = 0.016]. Mann-Whitney's *post-hoc* pairwise comparisons demonstrated that Grade 10 students had significantly higher EMST on laptops and computers than Grade 9 students on weekdays (MR Grade 9 = 141.80, Grade 10 = 175.10; *p* = 0.002) and weeknights (MR Grade 9 = 142.41, Grade 10 = 174.79; *p* = 0.003). Despite these statistical significances, the effect sizes were small (η^2^ = 0.029 and η^2^ = 0.027), indicating that the practical significance of these differences is modest. Similarly, Grade 10 students exhibited significantly higher EMST on smartphones on weekend days than Grade 9 students (MR Grade 9 = 144.60, Grade 10 = 173.70; *p* = 0.006), also with a small effect size (η^2^ = 0.023). However, Grade 9 students had significantly higher EMST on televisions during weekend days (MR Grade 9 = 184.11, Grade 10 = 153.94; *p* = 0.005), with a small effect size again (η^2^ = 0.023). These findings suggest a complex pattern of screen-based entertainment consumption between the grades, with variations that are statistically significant, but with modest practical implications.

Another Kruskal-Wallis *H*-test showed significant differences in adolescents' smartphone entertainment on weekdays [H (3) = 10.697, *p* = 0.014], weeknights [H (3) = 12.509, *p* = 0.006], as well as weekend days [H (3) = 11.377, *p* = 0.010]. Similarly, it revealed a significant variation in television entertainment on weekdays [H (3) = 11.872, *p* = 0.008]. Subsequent *post-hoc* comparisons demonstrated that Grade 11 students had significantly greater screen-based entertainment on smartphones on weekdays (MR Grade 9 = 137.69, Grade 11 = 165.88; *p* = 0.007), weeknights (MR Grade 9 = 132.15, Grade 11 = 168.89; *p* = 0.000), as well as weekend days (MR Grade 9 = 135.09, Grade 11 = 167.28; *p* = 0.002). Regardless of these significant differences, the effect sizes were small (η^2^ = 0.023, η^2^ = 0.039, and η^2^ = 0.032), indicating meaningful, yet not overwhelming practical significance. On the contrary, Grade 9 students spent significantly more EMST on televisions during weekdays than Grade 11 students (MR Grade 9 = 178.51, Grade 11 = 143.85; *p* = 0.001), but with a small effect size (η^2^ = 0.036), further highlighting the modest real-world implications although the difference was statistically significant.

A further Kruskal-Wallis *H*-test showed significant variations in the adolescents' weeknight screen-based entertainment on laptops and computers [H (3) = 11.355, *p* = 0.010] and weekend day screen-based entertainment on smartphones [H (3) = 11.377, *p* = 0.010], televisions [H (3) = 10.325, *p* = 0.016], tablets [H (3) = 9.294, *p* = 0.026], and also other screen media devices [H (3) = 9.881, *p* = 0.020]. Subsequent *post-hoc* comparisons between Grade 9 and Grade 12 students revealed that Grade 12 students had considerably higher entertainment screen time on laptops and computers during weeknights (MR Grade 9 = 133.40, Grade 12 = 160.26; *p* = 0.007) and on smartphones during weekend days (MR Grade 9 = 132.85, Grade 12 = 160.57; *p* = 0.005). Nevertheless, the effect sizes measured for these comparisons were still small (η^2^ = 0.024 and η^2^ = 0.026), indicating a modest practical significance. On the contrary, Grade 9 students reported more EMST on televisions (MR Grade 9 = 169.93, Grade 12 = 139.41; *p* = 0.003), tablets (MR Grade 9 = 166.33, Grade 12 = 141.47; *p* = 0.005), and also other screen media devices (MR Grade 9 = 165.28, Grade 12 = 142.07; *p* = 0.008) on weekend days. Again, the effect sizes were small (η^2^ = 0.029, η^2^ = 0.026, and η^2^ = 0.023), highlighting that these differences, although statistically meaningful, have no dramatic real-world effect.

Another Kruskal-Wallis *H*-test showed significant differences in the weekday EMST on smartphones [H (3) = 10.679, *p* = 0.014] and tablets [H (3) = 8.946, *p* = 0.030]. Similarly, it revealed a statistically meaningful difference in the weekend day EMST on television-connected devices [H (3) = 9.302, *p* = 0.026] as well as other screen media devices [H (3) = 9.881, *p* = 0.020]. *Post-hoc* pairwise comparisons demonstrated that Grade 11 students overtook their Grade 10 counterparts in the weekday smartphone EMST [MR Grade 10 = 195.50, Grade 11 = 226.69; *p* = 0.007], with a small effect size (η^2^ = 0.017). Comparisons between Grade 10 and Grade 12 showed that students in Grade 10 had significantly higher tablet-based EMST during weekdays and weeknights (MR Grade 10 = 218.01, Grade 12 = 190.15; *p* = 0.004 and MR Grade 10 = 216.50, Grade 12 = 191.87; *p* = 0.008), and also during weekend days through other screen media devices (MR Grade 10 = 217.95, Grade 12 = 190.22; *p* = 0.006). Yet, the effect sizes were small (η^2^ = 0.020, η^2^ = 0.016, and η^2^ = 0.018). Similarly, Grade 11 students reported significantly greater EMST than Grade 12 for their weeknight and weekend day on television-connected devices (MR Grade 11 = 209.68, Grade 12 = 183.59; *p* = 0.004 and MR Grade 11 = 211.18, Grade 12 = 182.00; *p* = 0.004), with small effect sizes (η^2^ = 0.021), suggesting modest real-world implications.

The findings revealed complex patterns in students' EMST across all timeframes and devices. During weekdays and weeknights, Grades 10 and 12 students had more EMST laptops and computers than their Grade 9 peers, while during weekdays and weekend days, Grade 10 and Grade 11 students used more smartphone entertainment. Compared to their upper-grade friends, Grade 9 students spent more entertainment time on television during weekend days. They also have higher EMST on tablets and other screen media devices, such as streaming devices and game consoles. Although these patterns were meaningful, the magnitude of effects was relatively modest across all tests. The observed patterns highlighted shifts in screen media preference for entertainment, with students in higher grades tending to spend more time on portable devices, while those in lower grade levels inclined to have lengthy entertainment on televisions.

As illustrated in Table 1, the Generalized Ordinal Logistic Regression Model (GOLRM) identified several significant predictors of adolescents' EMST across various devices and timeframes. Gender emerged as a critical factor, with male adolescents showing lower odds of high EMST on television-connected devices on weekdays (62% decrease, OR = 0.38, *p* = 0.000) and weeknights (49% decrease, OR = 0.51, *p* = 0.000). Conversely, they had higher odds of high EMST on tablets during weeknights (86% increase, OR = 1.86, *p* = 0.000), and on televisions during weekend days (63% increase, OR = 1.63, *p* = 0.036).

Parental employment status also played a significant role. Participants with unemployed mothers had 36% lower odds of high EMST on television-connected devices on weekdays (OR = 0.64, *p* = 0.025) and weekend days (OR = 0.64, *p* = 0.019). While fathers' unemployment was linked to higher odds of being in high weeknight EMST on these devices, this finding was not statistically significant (12% increase, OR = 1.12% higher odds, *p* = 0.76).

Parental education was another influential factor, with higher fathers' education associated with higher odds of adolescents' increased weekday and weekend day EMST using computers and laptops (31% rise, OR = 1.31, *p* = 0.024; 53% rise, OR = 1.53, *p* = 0.001, respectively). Conversely, mothers' education did not significantly correlate with adolescents' EMST on these devices during weekdays (10% increase, OR = 1.10, *p* = 0.41) and weekend days (2% decrease, OR = 0.98; *p* = 0.91).

Device-specific preference for EMST also varied by adolescents' age, grade level, and family size. Older adolescents had lower odds of high weekend day EMST on laptops and computers (33% decrease, OR = 0.67, *p* = 0.005), and an increase in their grade level was positively related to higher odds of high weekend day EMST on these devices (48% increase, OR = 1.48, *p* = 0.015). Furthermore, larger family sizes predicted higher weekend day EMST on televisions (55% rise, OR = 1.55, *p* = 0.002).

In sum, this study illuminates the impact of demographic, familial, and socioeconomic contexts on adolescents' EMST across various devices and timeframes. Male adolescents generally demonstrated lower weekday EMST on television-connected devices but higher tablet and television-based for entertainment during weeknights and weekend days. Mothers' unemployment was consistently related to lower EMST, while fathers' education was associated with higher EMST, particularly on computers. Grade levels and family sizes also influenced computer and television-based EMST. These findings suggest tailored interventions based on demographic, familial, and socioeconomic variations to promote balanced adolescent EMST across devices and timeframes.

## Discussion

This study explored adolescents' EMST, highlighting the complex interplay of demographic, familial, and socioeconomic factors affecting their entertainment consumption across different devices and timeframes. The findings provided a deeper understanding of adolescents' extended screen-based entertainment and its broader implications in life by integrating descriptive patterns with inferential insights.

Smartphones emerged as the dominant medium for adolescents' screen-based entertainment, with 64.6% (on weekdays), 64.8% (on weeknights), and 81.7% (on weekend days) exceeding the 2-h threshold of EMST on these devices. Among these adolescents, 28.5%, 27.2%, and 46.4% had over 4 h of daily entertainment on weekdays, weekdays, and weekend days, respectively. These findings are consistent with prior research emphasizing the pervasive role of smartphones in adolescents' entertainment lives, due to these devices' portable and versatile nature (Nagata et al., 2024; Rideout et al., 2010; Twenge et al., 2019). The study also revealed older adolescents' higher engagement in smartphone entertainment during weekdays (ρ = 0.110, *p* < 0.01). The grade level analyses further reinforced this with 10th, 11th, and 12th graders reporting higher EMST on smartphones than their grade 9 counterparts across all timeframes (see Table 8B). These findings corroborate research that stated the positive association between age and smartphone entertainment (Kardefelt-Winther et al., 2020; Rideout et al., 2022).

In contrast, television entertainment markedly declined, with one-third of adolescents reporting no entertainment on weekdays (33.1%) and weeknights (35.7%). However, weekend-day entertainment on this device remained notable, with 25.1% watching for 2–3 h per weekend day. These trends suggest a move from shared entertainment consumption to more individualized gadgets, like smartphones (Rideout et al., 2022; Twenge et al., 2019). The inverse correlation between age and weekend day television entertainment (ρ = −0.110, *p* < 0.01) further mirrored older adolescents' gravitation toward other screens that facilitate privacy, autonomy, and interaction, although earlier studies emphasize televisions' central role in family entertainment (Connell et al., 2015; Livingstone et al., 2017; Valkenburg et al., 2013). The divergence might suggest regional and cultural variations in adolescents' media preferences for entertainment.

Parental education also shaped adolescents' EMST patterns, with higher educational attainment positively correlating with adolescents' entertainment on laptops and computers (ρ = 0.141–0.159, *p* < 0.01). These findings align with Gentile et al. (2014) and Vandewater et al. (2005), who generally attributed this trend to greater resource accessibility in highly educated households. Nevertheless, the inverse association between paternal education and adolescents' television entertainment (ρ = −0.073, *p* < 0.05) may suggest device-specific parental mediation, whereby better-educated parents discourage extended entertainment on passive media in favor of alternative activities. However, this finding contradicts (Livingstone et al., 2017), who emphasized the restrictive role of parents with more education by reducing their children's overall screen-based entertainment. The inconsistencies between the present and earlier studies may suggest the importance of sociocultural and regional dynamics in adolescents' entertainment consumption.

Similarly, parental employment affected adolescents' EMST. Adolescents with unemployed mothers had lower odds of weekday entertainment on television-connected devices (36% decrease, OR = 0.64, *p* = 0.025), suggesting higher parental supervision or inaccessibility of these devices due to economic constraints. However, Carlson et al. (2008), Lauricella et al. (2015), Rideout et al. (2022), and (Carlson et al., 2008) posit that parental unemployment can foster an unstructured household environment, potentially turning adolescents' attention to higher screen-based entertainment.

Gender differences further highlighted the complex nature of adolescents' EMST. Male adolescents were over-represented for tablets across all timeframes, as shown in their higher odds of using these devices for entertainment (86% increase, OR = 1.86, *p* = 0.000, for both the weekday and weeknight EMST categories; 72% increase, OR = 1.72, *p* = 0.001, for the weekend day category). This finding supports (Rehbein et al., 2016) who observed that males resort to more interactive media platforms, possibly for gaming. Conversely, females demonstrated more entertainment on televisions (ρ = 0.095, *p* < 0.05), reflecting a predisposition toward shared media. Interestingly, the absence of significant gender differences in using smartphones for extended entertainment consumption challenges earlier studies, such as Vorderer et al. (2016), suggesting a convergence between genders, may be due to the device's convenience for entertainment.

The theoretical implications of these findings are underpinned by the uses and gratifications framework (Katz et al., 1974), which posits that user motivations and needs drive media selection. For example, older adolescents' greater tendency for smartphone entertainment suggests a desire for privacy and autonomy, as highlighted in a study (Twenge and Campbell, 2018). Moreover, the Ecological Systems Theory (Bronfenbrenner, 1981) contextualizes the influence of family size and socioeconomic factors on EMST, as larger families in this study, were correlated with higher weekend days' television entertainment (ρ = 0.098, *p* < 0.01), reflecting the communal nature of television entertainment, as also noted by Crawford (2020). This finding illuminates the importance of familial contexts in mediating device preferences for entertainment. Moreover, most adolescents' (64.6%) extended engagement in smartphone entertainment during weekend days corroborates the Media Affordance Theory which assumes devices' nature, such as multifunctionality and ease of use in shaping consumption patterns (Evans et al., 2017).

In summary, this study underscores the role of screen-based devices in adolescents' extended entertainment consumption across different timeframes. Moreover, adolescents' entertainment screen time is shaped by various factors, including demographic, familial, and socioeconomic conditions. Given the implications of excessive screen-based entertainment in adolescents' lives, these findings point to the importance of interventions to correct the imbalances in EMST.

## Conclusion

The study revealed complex patterns of adolescents' excessive EMST across devices and timeframes. Smartphones were the most extensively used screen devices for entertainment, among adolescents across all timeframes. In contrast, although televisions were once the dominant form of entertainment media, their use has shown a marked decline, especially on weekdays and weeknights, reflecting a shift toward more individualized and portable screens like smartphones. Laptop and computer-based entertainment remained consistently high across all timeframes, although this was comparatively lower than smartphone entertainment. The lowest level of engagement in extended EMST was on tablets, TV-connected devices (e.g., streaming devices and game consoles), and other screen media devices, with relatively higher consumption on weekend days.

Age, gender, grade level, parental employment, parental education, and family size emerged as significant factors influencing adolescents' excessive EMST across devices and timeframes. The transition toward more personalized portable screens, such as smartphones, for prolonged entertainment consumption was particularly evident among older adolescents and higher-grade students. At the same time, younger adolescents and those from larger families gravitated toward shared media like televisions. Parental employment and education distinctly shaped adolescents' EMST patterns, highlighting the central role of familial and socioeconomic contexts in adolescents' screen-based entertainment. Gendered trends persisted, especially in consuming extended entertainment on tablets among male adolescents, while extensive engagement in television entertainment was notably high among females and younger adolescents. Hitherto, smartphone entertainment transcended those gender divides. The effect size across all the associations was small. Overall, the findings suggest a need for tailored interventions to promote balanced EMST while accounting for the complex interplay of these factors affecting adolescents' screen-based entertainment.

These findings contribute to the literature on media studies, particularly regarding adolescents' screen-based entertainment. Policymakers and health professionals should raise awareness about the effects of prolonged screen-based entertainment on academic performance and sleep, promoting balanced engagement with such content. Schools should develop curricula that support digital wellbeing and offer active recreational options to reduce heavy reliance on screens for entertainment. By implementing these recommendations, we can foster healthier screen time habits among adolescents, encouraging more balanced and beneficial interactions with screen devices.

## Limitations

The use of a cross-sectional research design in this study poses challenges in establishing causal relationships. Additionally, reliance on self-reported data may introduce bias. Future research should consider longitudinal studies that incorporate qualitative methods to contextualize quantitative findings. Exploring sociocultural dynamics can offer deeper insights into the contextual factors influencing adolescents' engagement with screen-based entertainment. Furthermore, integrating input from stakeholders will lead to a more comprehensive understanding of this issue.

## Data Availability

The original contributions presented in the study are included in the article/supplementary material, further inquiries can be directed to the corresponding author.

## References

[B1] Addis Ababa City Administration Education Bureau (2023). Educational Statistics Annual Abstract (2021/2022). Available online at: http://moe.gov.et/resources/annual-abstract/4 (Accessed September 05, 2024).

[B2] Agresti A. (2010). Analysis of Ordinal Categorical Data, 2nd Edn. Wiley.

[B3] Akoglu H. (2018). User's guide to correlation coefficients. Turk. J. Emerg. Med. 18, 91–93. 10.1016/j.tjem.2018.08.001 30191186 PMC6107969

[B4] Bagot K. Tomko R. Marshall A. T. Hermann J. Cummins K. Ksinan A.. (2022). Youth screen use in the ABCD^®^ study. Dev. Cogn. Neurosci. 57:101150. 10.1016/j.dcn.2022.101150 36084446 PMC9465320

[B5] Beyens I. Pouwels J. L. Van Driel I. I. Keijsers L. Valkenburg P. M. (2020). The effect of social media on well-being differs from adolescent to adolescent. Sci. Rep. 10:10763. 10.1038/s41598-020-67727-7 32612108 PMC7329840

[B6] Bronfenbrenner U. (1981). The Ecology of Human Development: Experiments by Nature and Design. Harvard University Press. 10.2307/j.ctv26071r6

[B7] Camerini A.-L. Gerosa T. Marciano L. (2021). Predicting problematic smartphone use over time in adolescence: a latent class regression analysis of online and offline activities. New Media Soc. 23, 3229–3248. 10.1177/1461444820948809

[B8] Carlson S. A. Fulton J. E. Lee S. M. Maynard L. M. Brown D. R. Kohl H. W.. (2008). Physical education and academic achievement in elementary school: data from the early childhood longitudinal study. Am. J. Public Health 98 721–727. 10.2105/AJPH.2007.117176 18309127 PMC2377002

[B9] Cochran W. (1977). Sampling Techniques, 3rd Edn. New York, NY: Wiley.

[B10] Cohen J. (1988). Statistical Power Analysis for the Behavioral Sciences, 2nd Edn. New York: L. Erlbaum Associates.

[B11] Connell S. L. Lauricella A. R. Wartella E. (2015). Parental Co-use of media technology with their young children in the USA. J. Child. Media 9, 5–21. 10.1080/17482798.2015.99744028791572

[B12] Conover W. J. Conover W. J. (1999). Practical Nonparametric Statistics, 3rd Edn. New York: Wiley.

[B13] Council on Communications and Media (2016). Media use in school-aged children and adolescents. Pediatrics 138:e20162592. 10.1542/peds.2016-2592 27940794

[B14] Council on Communications Media Strasburger V. C. Hogan M. J. Mulligan D. A. Ameenuddin N. Christakis D. A.. (2013). Children, adolescents, and the media. Pediatrics 132, 958–961. 10.1542/peds.2013-2656 28448255

[B15] Crawford M. (2020). Ecological systems theory: exploring the development of the theoretical framework as conceived by Bronfenbrenner. J. Public Health Issues Pract. 4:170. 10.33790/jphip1100170

[B16] Creswell J. W. (2003). Research Design: Qualitative, Quantitative, and Mixed Method Approaches, 2nd Edn. Thousand Oaks, CA: Sage Publications.

[B17] Creswell J. W. (2008). Research Design: Qualitative, Quantitative, and Mixed Methods Approaches, 2nd Edn. Thousand Oaks, CA: Sage Publications.

[B18] Evans S. K. Pearce K. E. Vitak J. Treem J. W. (2017). Explicating affordances: a conceptual framework for understanding affordances in communication research: explicating affordances. J. Comput. Mediat. Commun. 22, 35–52. 10.1111/jcc4.12180

[B19] Field A. (2017). Discovering Statistics Using IBM SPSS Statistics, 5th Edn. SAGE Publications.

[B20] Fowler F. J. (2014). Survey Research Methods, 4th Edn. SAGE.

[B21] Fullerton A. S. Xu J. (2016). Ordered Regression Models: Parallel, Partial, and Non-Parallel Alternatives. New York: Chapman and Hall/CRC.

[B22] Gentile D. A. Reimer R. A. Nathanson A. I. Walsh D. A. Eisenmann J. C. (2014). Protective effects of parental monitoring of children's media use: a prospective study. JAMA Pediatr. 168:479. 10.1001/jamapediatrics.2014.146 24686493

[B23] Groves R. M. Peytcheva E. (2008). The impact of nonresponse rates on nonresponse bias: a meta-analysis. Public Opin. Quart. 72, 167–189. 10.1093/poq/nfn011

[B24] Hair J. F. (Ed.). (2010). Multivariate Data Analysis: A Global Perspective, 7th Edn. Upper Saddle River, NJ: Pearson Education.

[B25] Hollander M. Wolfe D. A. Chicken E. (2014). Nonparametric Statistical Methods, 3rd Edn. John Wiley and Sons, Inc.

[B26] Howell D. C. (2013). Statistical Methods for Psychology, 8th Edn. Wadsworth: Cengage Learning.

[B27] Huang C. (2018). Social network site use and academic achievement: a meta-analysis. Comput. Educ. 119, 76–83. 10.1016/j.compedu.2017.12.010

[B28] Kardefelt-Winther D. Rees G. Livingstone S. (2020). Contextualising the link between adolescents' use of digital technology and their mental health: a multi-country study of time spent online and life satisfaction. J. Child Psychol. Psychiatr. 61, 875–889. 10.1111/jcpp.13280 32634259

[B29] Katz E. Blumler J. Gurevitch M. (1974). The Uses of Mass Communications: Current Perspectives on Gratifications Research, Vol. 3. Beverly Hills: Sage Annual Reviews of Communication Research.

[B30] Keles B. McCrae N. Grealish A. (2020). A systematic review: the influence of social media on depression, anxiety and psychological distress in adolescents. Int. J. Adolesc. Youth 25, 79–93. 10.1080/02673843.2019.1590851

[B31] Lauricella A. R. Wartella E. Rideout V. J. (2015). Young children's screen time: the complex role of parent and child factors. J. Appl. Dev. Psychol. 36, 11–17. 10.1016/j.appdev.2014.12.001

[B32] Levin K. A. (2006). Study design III: cross-sectional studies. Evid. Based Dent. 7, 24–25. 10.1038/sj.ebd.6400375 16557257

[B33] Liu I. Agresti A. (2005). The analysis of ordered categorical data: an overview and a survey of recent developments. Test 14, 1–73. 10.1007/BF02595397

[B34] Livingstone S. Davidson J. Bryce J. (2017). Children's online activities, risks, and safty: lliterature review. J. Commun. 68, 1–35.

[B35] Livingstone S. Helsper E. (2007). Gradations in digital inclusion: children, young people and the digital divide. New Media Soc. 9, 671–696. 10.1177/1461444807080335

[B36] Livingstone S. Mascheroni G. Staksrud E. (2018). European research on children's internet use: assessing the past and anticipating the future. New Media Soc. 20, 1103–1122. 10.1177/1461444816685930

[B37] Lohr S. L. (2022). Sampling: Design and Analysis, 3rd Edn. New York: CRC Press.

[B38] Long J. S. Freese J. (2014). Regression Models for Categorical Dependent Variables using Stata, 3rd Edn. College Station, TX: Stata Press.

[B39] Madigan S. McArthur B. A. Anhorn C. Eirich R. Christakis D. A. (2020). Associations between screen use and child language skills: a systematic review and meta-analysis. JAMA Pediatr. 174:665. 10.1001/jamapediatrics.2020.0327 32202633 PMC7091394

[B40] McCullagh P. (1980). Regression models for ordinal data. J. Royal Stat. Soc. 42, 109–127. 10.1111/j.2517-6161.1980.tb01109.x

[B41] McDonald J. (2014). Handbook of Biological Statistica, 3rd Edn. Baltimor, MD: Sparky House Publishing.

[B42] McKight P. E. Najab J. (2010). Kruskal-Wallis test. Corsini Encycl. Psychol. 1, 1–10. 10.1002/9780470479216.corpsy0491

[B43] Montgomery D. C. Peck E. A. Vining G. G. (2020). Introduction to Linear Regression Analysis, 5th Edn. Wiley.

[B44] Nagata J. M. Paul A. Yen F. Smith-Russack Z. Shao I. Y. Al-shoaibi A. A. A.. (2024). Associations between media parenting practices and early adolescent screen use. Pediatr. Res. 97, 403–410. 10.1038/s41390-024-03243-y 38834780 PMC11626836

[B45] Nikken P. Schols M. (2015). How and why parents guide the media use of young children. J. Child Family Stud. 24, 3423–3435. 10.1007/s10826-015-0144-4 26472932 PMC4598347

[B46] Nunnally J. Bernstein I. (1994). Psychometric Theory, 3rd Edn. New York, NY: McGraw-Hill.

[B47] Peterson B. Harrell F. E. (1990). Partial proportional odds models for ordinal response variables. Appl. Stat. 39:205. 10.2307/2347760

[B48] Priftis N. Panagiotakos D. (2023). Screen time and its health consequences in children and adolescents. Children 10:1665. 10.3390/children10101665 37892328 PMC10605067

[B49] Przybylski A. K. Weinstein N. (2019). Digital screen time limits and young children's psychological well-being: evidence from a population-based study. Child Dev. 90, e56–e65. 10.1111/cdev.13007 29235663

[B50] Rehbein F. Staudt A. Hanslmaier M. Kliem S. (2016). Video game playing in the general adult population of Germany: can higher gaming time of males be explained by gender specific genre preferences? Comput. Hum. Behav. 55, 729–735. 10.1016/j.chb.2015.10.016

[B51] Rideout V. J. Foehr U. Roberts D. (2010). M2: Media in the Lives of 8-to-18-year-Olds. Menlo Park, CA: Henry J. Kaiser Family Foundation.

[B52] Rideout V. J. Peebles A. Mann S. Robb M. (2022). The Common Senes Census: Media Use by Tweens and Teens, 2021. Common Senes Media.

[B53] Rosen L. D. Whaling K. Carrier L. M. Cheever N. A. Rokkum J. (2013). The media and technology usage and attitudes scale: an empirical investigation. Comput. Hum. Behav. 29, 2501–2511. 10.1016/j.chb.2013.06.006 25722534 PMC4338964

[B54] Schmidt-Persson J. Rasmussen M. G. B. Sørensen S. O. Mortensen S. R. Olesen L. G. Brage S.. (2024). Screen media use and mental health of children and adolescents: a secondary analysis of a randomized clinical trial. JAMA Netw. Open 7:e2419881. 10.1001/jamanetworkopen.2024.19881 38995646 PMC11245724

[B55] Sheskin D. J. (2020). Handbook of Parametric and Nonparametric Statistical Procedures, 5th Edn. New York: Chapman and Hall/CRC.

[B56] Shi X. Chen L. Uher C. (2016). Recent advances in high-performance bulk thermoelectric materials. Int. Mater. Rev. 61, 379–415. 10.1080/09506608.2016.1183075

[B57] Siegel S. Castellan N. J. (2003). Nonparametric Statistics for the Behavioral Sciences, 2nd Edn. New York: McGraw-Hill.

[B58] Tavakol M. Dennick R. (2011). Making sense of Cronbach's alpha. Int. J. Med. Educ. 2, 53–55. 10.5116/ijme.4dfb.8dfd 28029643 PMC4205511

[B59] Throuvala M. A. Griffiths M. D. Rennoldson M. Kuss D. J. (2019). A ‘Control Model' of social media engagement in adolescence: a grounded theory analysis. Int. J. Environ. Res. Public Health 16:4696. 10.3390/ijerph16234696 31775387 PMC6926519

[B60] Turkle S. (2015). Reclaiming Conversation: The Power of Talk in a Digital Age. New York, NY: Penguin Publishing Group.

[B61] Twenge J. M. Campbell W. K. (2018). Associations between screen time and lower psychological well-being among children and adolescents: evidence from a population-based study. Prevent. Med. Rep. 12, 271–283. 10.1016/j.pmedr.2018.10.003 30406005 PMC6214874

[B62] Twenge J. M. Martin G. N. Spitzberg B. H. (2019). Trends in U.S. adolescents' media use, 1976–2016: the rise of digital media, the decline of TV, and the (near) demise of print. Psychol. Pop. Media Cult. 8, 329–345. 10.1037/ppm0000203

[B63] Ursachi G. Horodnic I. A. Zait A. (2015). How reliable are measurement scales? External factors with indirect influence on reliability estimators. Procedia Econ. Finance 20, 679–686. 10.1016/S2212-5671(15)00123-927885969

[B64] Valkenburg P. M. Piotrowski J. T. Hermanns J. De Leeuw R. (2013). Developing and validating the perceived parental media mediation scale: a self-determination perspective: parental mediation scale. Hum. Commun. Res. 39, 445–469. 10.1111/hcre.12010

[B65] Vandewater E. A. Park S.-E. Huang X. Wartella E. A. (2005). “No—You Can't Watch That”: parental rules and young children's media use. Am. Behav. Sci. 48, 608–623. 10.1177/0002764204271497

[B66] Vandewater E. A. Rideout V. J. Wartella E. A. Huang X. Lee J. H. Shim M. (2007). Digital childhood: electronic media and technology use among infants, toddlers, and preschoolers. Pediatrics 119, e1006–e1015. 10.1542/peds.2006-1804 17473074

[B67] Vizcaino M. Buman M. DesRoches C. T. Wharton C. (2019). Reliability of a new measure to assess modern screen time in adults. BMC Public Health 19:1386. 10.1186/s12889-019-7745-6 31660931 PMC6816215

[B68] Vorderer P. Krömer N. Schneider F. M. (2016). Permanently online – permanently connected: explorations into university students' use of social media and mobile smart devices. Comput. Hum. Behav. 63, 694–703. 10.1016/j.chb.2016.05.085

[B69] Wang X. Cheng Z. (2020). Cross-sectional studies. Chest 158, S65–S71. 10.1016/j.chest.2020.03.012 32658654

[B70] Williams R. (2006). Generalized ordered logit/partial proportional odds models for ordinal dependent variables. Stata J. 6, 58–82. 10.1177/1536867X0600600104

[B71] Yamane T. (1967). Statistics, an Introductory Analysis, 2nd Edn. New York: Harber and Row.

[B72] Zablotsky B. Arockiaraj B. Haile G. Ng A. E. (2024). Daily screen time among teenagers: United States, July 2021 – December 2023. NCHS Data Brief 513:CS354544. 10.15620/cdc/168509 39729081 PMC11701796

[B73] Zar J. H. (2014). Biostatistical Analysis (Fifth Edition, Pearson new international edition). Upper Saddle River, NJ: Pearson Education Limited.

